# Behavioral Characterization of the Hyperphagia Synphilin-1 Overexpressing Mice

**DOI:** 10.1371/journal.pone.0091449

**Published:** 2014-05-14

**Authors:** Xueping Li, Yada Treesukosol, Alexander Moghadam, Megan Smith, Erica Ofeldt, Dejun Yang, Tianxia Li, Kellie Tamashiro, Pique Choi, Timothy H. Moran, Wanli W. Smith

**Affiliations:** 1 Department of Pharmaceutical Sciences, University of Maryland School of Pharmacy, Baltimore, Maryland, United States of America; 2 Department of Psychiatry, Johns Hopkins University School of Medicine, Baltimore, Maryland, United States of America; Sapienza University of Rome, Italy

## Abstract

Synphilin-1 is a cytoplasmic protein that has been shown to be involved in the control of energy balance. Previously, we reported on the generation of a human synphilin-1 transgenic mouse model (SP1), in which overexpression of human synphilin-1 resulted in hyperphagia and obesity. Here, behavioral measures in SP1 mice were compared with those of their age-matched controls (NTg) at two time points: when there was not yet a group body weight difference (“pre-obese”) and when SP1 mice were heavier (“obese”). At both time points, meal pattern analyses revealed that SP1 mice displayed higher daily chow intake than non-transgenic control mice. Furthermore, there was an increase in meal size in SP1 mice compared with NTg control mice at the obese stage. In contrast, there was no meal number change between SP1 and NTg control mice. In a brief-access taste procedure, both “pre-obese” and “obese“ SP1 mice displayed concentration-dependent licking across a sucrose concentration range similar to their NTg controls. However, at the pre-obese stage, SP1 mice initiated significantly more trials to sucrose across the testing sessions and licked more vigorously at the highest concentration presented, than the NTg counterparts. These group differences in responsiveness to sucrose were no longer apparent in obese SP1 mice. These results suggest that at the pre-obese stage, the increased trials to sucrose in the SP1 mice reflects increased appetitive behavior to sucrose that may be indicative of the behavioral changes that may contribute to hyperphagia and development of obesity in SP1 mice. These studies provide new insight into synphilin-1 contributions to energy homeostasis.

## Introduction

Synphilin-1 (919 aa) is a cellular protein predominantly expressed in the cytosol [1]. Synphilin-1 protein is present in many tissues with enriched expression in neurons [1]. Synphilin-1 has been reported to interact with a number of proteins including alpha-synuclein, parkin and other proteasome/ubiquitin associated proteins [1]–[5]. Previous reports showed that synphilin-1 enhances the formation of intracellular protein inclusions and may be involved in Parkinson’s disease (PD) pathogenesis [1]–[4], [6]. Synphilin-1 can reduce PD-linked mutant alpha-synuclein-, rotenone-, and 6-HODA-induced toxicity in vitro and delays alpha-synucleinopathies in a PD mouse model in vivo [7], [8]. Recent studies of human synphilin-1 transgenic *Drosophila* and mouse models have revealed that overexpression of human synphilin-1 results in increases in food intake, body weight and fat deposition, resembling key features of human obesity [9], [10]. While these studies suggest a role for synphilin-1 in regulating energy balance, the biological mechanisms underlying synphilin-1-mediated hyperphagia and obesity are unknown.

Hyperphagia is a core feature of many obesity models and alterations in multiple signaling pathways can contribute to hyperphagia [11]–[17]. Thus, detailed analysis of food intake changes in the SP1 mouse model could provide insights into the underlying mechanisms that are driving the hyperphagia [9]. Increased food intake can be result from increased meal size, meal number, or both [11]–[17]. The direct controls of meal size can be categorized into positive and negative signals that maintain and terminate eating behavior respectively [18]. Positive feedback is elicited by stimulation of gustatory, olfactory, and somatosensory receptors in the oral cavity whereas negative feedback is produced by contact with receptors in the oral cavity, stomach and small intestine [19], [20]. Increased orosensory stimulation and/or reduced sensitivity to postingestive inhibitory signals would alter meal pattern resulting in increases in food intake.

In the present studies, we tested separate cohorts of 6–8-week–old (“pre-obese) and 4-month-old (“obese”) SP1 male mice and age-matched NTg controls to assess 1) how meal pattern parameters change to reflect the increased food intake in SP1 mice, 2) whether synphilin-1 expression alters appetitive behaviors or unconditioned licking responses to sucrose that differed from those of NTg control mice, and 3) whether there are developmental feeding behavior changes related to hyperphagia and obesity. The results from these behavioral assessments would provide an increased understanding of how synphilin-1-expression results in hyperphagia and obesity.

## Materials and Methods

### Subjects

The SP1 mice that expressed human synphilin-1 in neurons under the mouse prion protein promoter were generated as described previously [9]. SP1 mice for behavioral experiments were generated by successive backcrossing with the C57BL6 strain. At 3 weeks of age, SP1 mice and age-matched controls (NTg) were weaned. Then, PCR-genotyping was performed to separate non-transgenic and SP1 mice as described previously [7]. Briefly, a 1 cM tail tip of each mouse was cut and subjected to DNA extraction. Then the resulting DNA was subjected to PCR using primers to detect the synphilin-1 sequences. Male NTg and SP1 mice at 6–10 weeks (“pre-obese”) and 4 months (“obese”) of age were used as subjects in the behavioral procedures described. All animal experiments were approved by the Johns Hopkins University Institutional Animal Care and Use Committee.

### Western Blot Analysis

The brains of Synphilin-1 and NTg control mice were homogenated as described previously [9]. The brain homogenates were subjected to western blot analysis using anti-synphilin-1 antibodies as described previously [9].

### Meal Pattern Analysis

Mice for the “pre-obese” comparisons (n = 9–10/group) were single-housed in DietMax System food intake monitoring cages (AccuScan Instruments, Inc., Columbus, OH) (length 32 cm, width 22 cm) with *ad libitum* access to powdered chow (6% fat, 3.1 kcal/g; 2018 Tekland, Harlan) and water. Food intake was monitored continuously over 23-h daily test sessions as previously described [21]. Powdered laboratory chow diet was provided *ad libitum* in a food jar placed on a scale in the feeding compartment of the cage. The animals had access to the food jar via an opening in the wall of the cage. A water bottle was mounted on an adjacent wall of the cage. Water was available *ad libitum*.

Testing began after 7 days of habituation to the experimental environment and maintenance on chow diet. Meal pattern measures to powdered chow were taken for three consecutive days. For the 4–6 weeks old mice, intake of the powdered high fat diet (45% fat, 4.73 kcal/g; D12451, Research Diets) was measured for the next 3 consecutive days A feeding bout was operationally defined as requiring ≥0.02 g food. An interval of ≥10 min without food intake defined the termination of a meal. This bout criteria on average accounted for ∼90% of the feeding data in the current study. One mouse produced excessive spillage of the standard chow thus data from that animal were excluded for data analysis.

For the “obese” comparisons (n = 6–7/group), mice were single-housed under similar test conditions but in test cages (Coulbourn Instruments, Allentown PA) (19 cm×19 cm) equipped with a pellet dispenser that delivers 20-mg chow pellets (3.8% fat, 3.35 kcal/g; Bioserve) as previously described [22]. Removal of a pellet from the feeding dish activated the pellet dispenser to deliver another pellet. Meal pattern parameters were measured across four consecutive days. A meal was operationally defined as at least 3 pellets preceded and followed by at least 10 min without food intake.

### Brief-access Taste Procedure

This behavior assessment was performed as previously described with slight modification [23]. Additional cohorts of male mice at 6–10 weeks (n = 8/group) or 4 months of age (n = 8/group) were individually housed in a procedure room where humidity, temperature, and a 12 h light-12 h dark cycle were automatically controlled. Behavioral testing started after at least 3 days of adaption to the experimental procedure room. The mice were on a water-restriction schedule during behavioral training. Water was taken away from the home cages for 23 hours before testing. Animals were only allowed access to water during the training sessions. After the last training session, animals were allowed free access water in the home cages. Mice were then tested with different sucrose concentrations under a partial food and water restriction condition in which they were presented ∼1 g of chow and ∼2 ml of water for ∼23 hours before testing. At least one repletion day (free access to water and chow) followed each testing day under food and water restriction.

Training and testing was performed in a lickometer (Davis MS-160, DiLog Instruments, Tallahassee FL) during the light cycle as described previously [23], [24]. The animal was put in the testing chamber and had access to a single spout positioned about 5 mm behind a slot. A potential trial was cued with the opening of a shutter exposing the drinking spout. The mouse licked the spout to initiate a trial. The shutter closed after each trial (5 s). During each intertrial interval (8 s), the tube presentation was changed by a motorized block, and then the shutter re-opened for the next trial. Animals were allowed to initiate as many trials as possible during the 25-min sessions. Concentrations were presented in randomized blocks of 7. Water and six concentrations of sucrose (0.03, 0.06, 0.15, 0.3, 0.6 and 1.0 M) that cover the dynamic range of responsiveness for mice were chosen. All solutions were prepared daily using distilled water.

Animals were subjected to a ∼23 h water-restriction schedule for the four days of behavioral training during which animals were allowed free access water only during the daily sessions. On days 1 and 2, animals were allowed access a stationary spout of water for 30-min sessions. On days 3 and 4, animals were allowed access to seven spouts of water in 5-s trials across 25-min sessions. After the end of session 4, animals were allowed free access water in their home cages. The next week, animals were subjected to the tests with water and six concentrations of sucrose across three 25-min daily sessions under a partial water and food restriction condition with one repletion day interspersed between testing days.

For each sucrose concentration, the mean number of licks was determined by all trials across the three test sessions. A Licks Relative to Water Value was calculated by subtracting the mean number of licks to water from the mean number of licks at each concentration as described previously [25]–[29]. This method provided concentration-response curves that were adjusted to a water baseline. The values for each concentration were compared using two-way repeated measures analyses of variance (ANOVA). If an individual animal did not initiate at least 2 trials per concentration collapsed across the three sucrose testing sessions, this animal was excluded from the Licks Relative to Water Value analysis. Data from all animals were included for analysis of number of trials. Two-sample t-tests were used to compare body weight, mean licks to a stationary spout of water, Interlick Interval (ILI) Values, number of trials initiated and chow intake across the two groups. Only ILIs that were between 70 and 200 ms were subjected to analysis given that ILI values less than 70 ms were defined as double licks and values more than 200 ms were considered pauses between licking bursts [25]. A p<0.05 was considered statistical significant for all analyses. The curves were fit to data using a previously described logistic function [23]: f(x) = a/(1+10^(x-c)b^), where x = log_10_ stimulus concentration, a = asymptotic lick response, b = slope and c = log_10_ concentration at the inflection point.

## Results

### Average Meal Size is Increased in SP1 Mice

**Figure 1:**
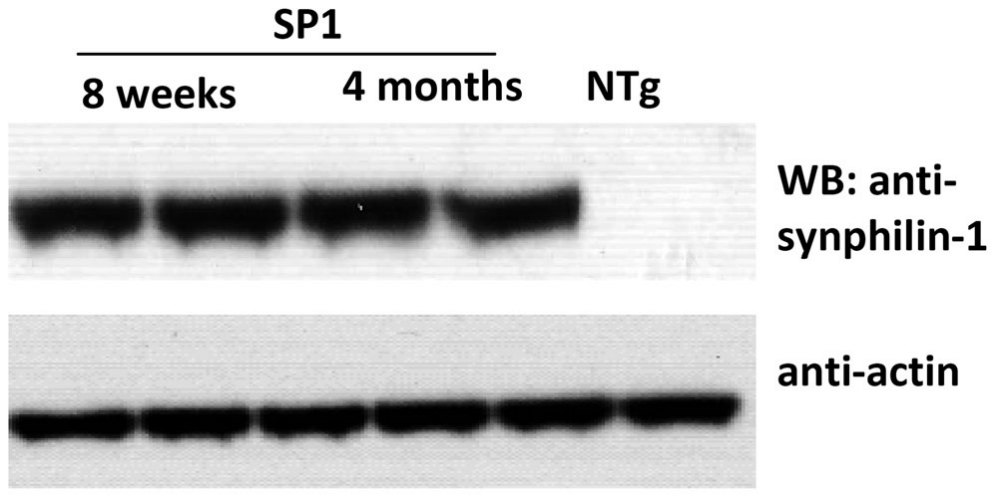
Human synphilin-1 expression in brains of SP1 mice. Brain homogenates from SP1 and non-transgenic control mice were subjected to western blot analysis using anti-synphilin-1 and anti-actin antibodies.

The cohorts of both pre-obese (6–10 weeks of age) and obese (4 months of age) SP1 transgenic mice were generated as described previously [9]. Human synphilin-1 proteins were expressed in brains at both pre-obese and obese stages as determined by western blot analysis using anti-human synphilin-1 antibodies (Figure 1).

**Figure 2:**
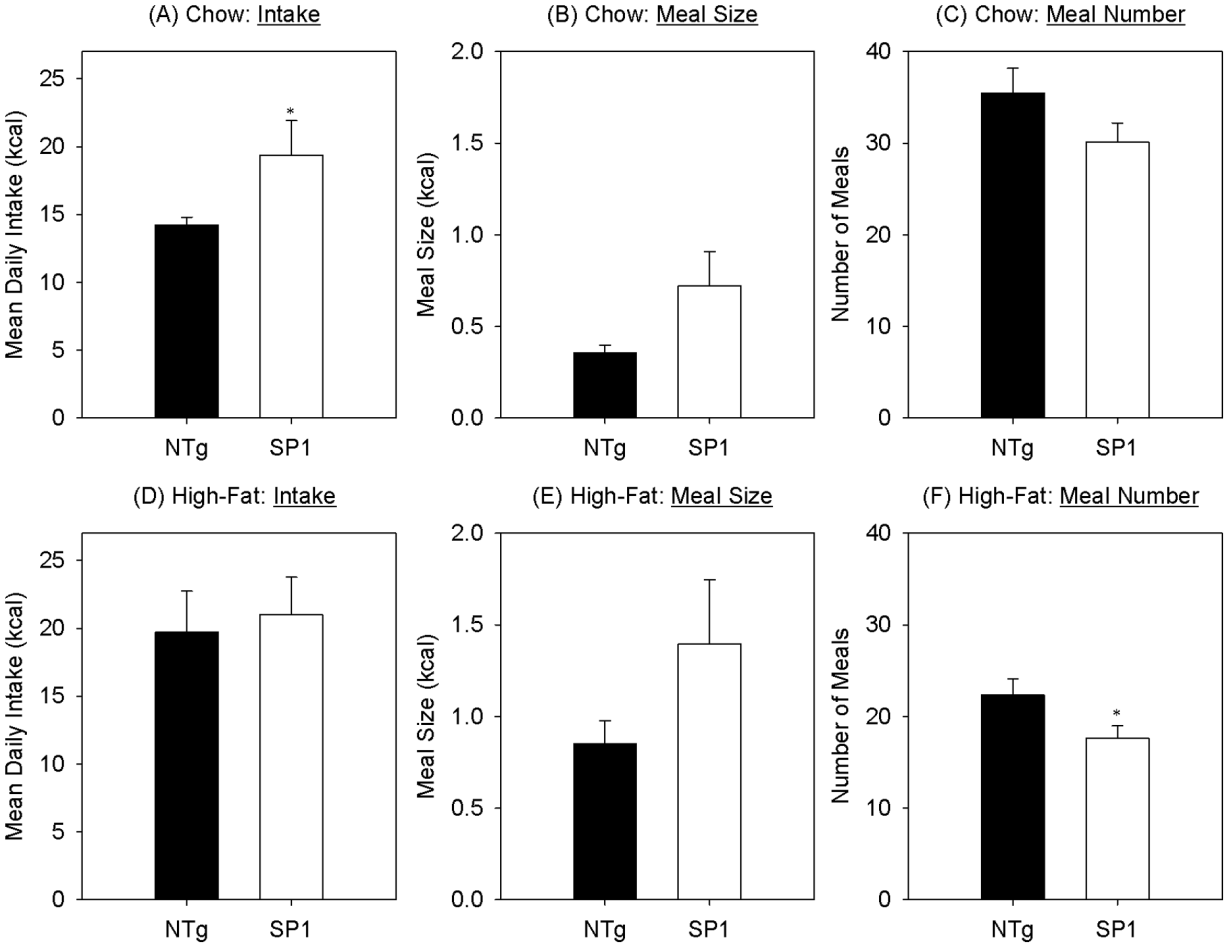
Meal pattern of pre-obese SP1 mice. Mean (A) daily intake, (B) meal size and (C) meal number for standard chow and (D) intake, (E) meal size and (F) meal number for high-fat diet for NTg (black bars) synphilin-1 mice (white bars) at the “pre-obese” time-point. *p<0.05, compared with non-transgenic mice.

At the pre-obese time point, there was no significant group difference in body weights between SP1 and NTg mice. However, SP1 mice consumed significantly more standard chow than did controls mice {Figure 2A; t(17) = −2.461, p = 0.025}. Meal pattern assessment showed that there was a trend for an increase in meal size (Figure 2B) and a slight decrease in meal number (Figure 2C) although these data did not reach statistical differences in either meal size {t(17) = −1.981, p = 0.064} or meal number {Figure 2C; t(17) = 1.540, p = 0.142}.

When animals were switched to a high-fat diet, a statistical difference in daily intake between the SP-1 and NTg mice was no longer apparent {Figure 2D; t(18) = −0.314, p = 0.757}. Two-sample t-tests did not reveal a significant group difference in meal size {Figure 2E; t(18) = −1.453, p = 0.163} but SP1 mice initiated fewer meals than NTg controls {Figure 2F; (t) = 2.102, p = 0.05}. Paired t-tests revealed that both groups increased meal size when fed high-fat diet, SP1 {t(8) = −2.773, p = 0.024]], NTg {t(9) = −4.534, p = 0.001}. There was also a significant decrease in meal number {SP1 (t(8) = 7.858, p<0.001}, NTg {t(9) = 5.213, p = 0.001} in both groups when switched from chow to the high-fat diet such that this did not result in a significant overall difference in intake for either group {SP1 (t(8) = 0.217, p = 0.833}, NTg {t(9) = −1.782, p = 0.108}.

**Figure 3:**
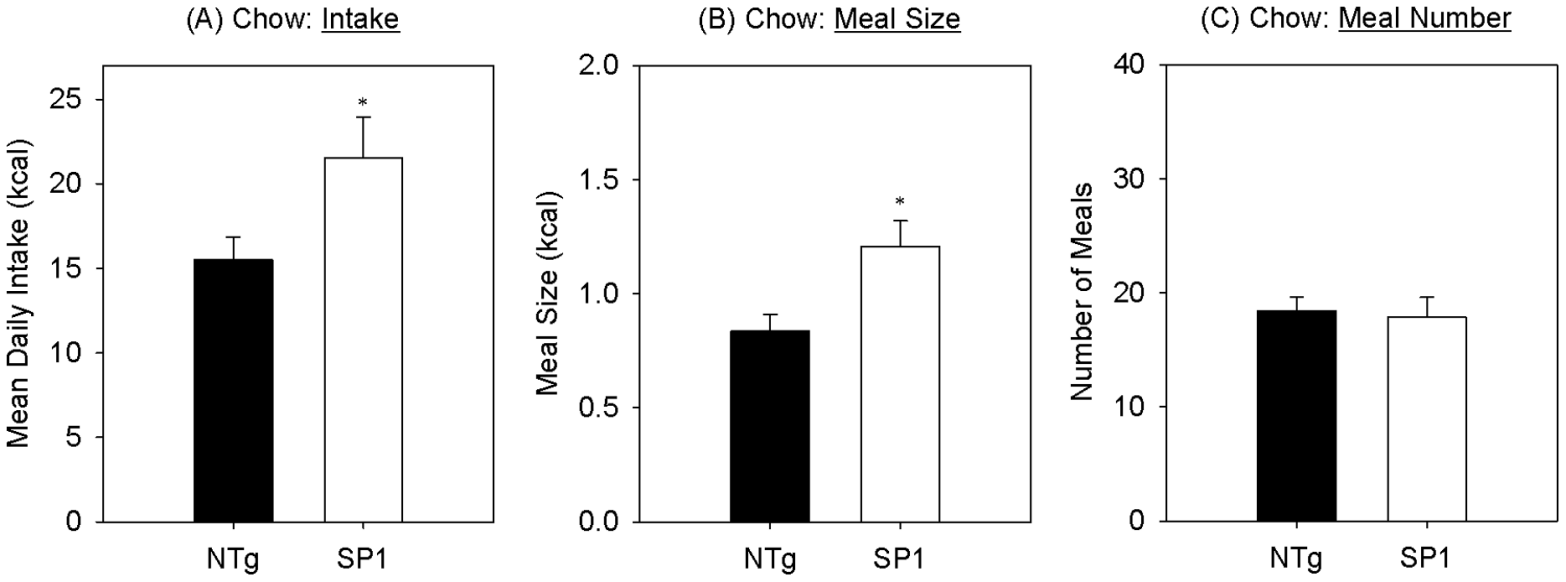
Meal pattern of SP1 obese mice. A. body weight. Mean (B) daily intake, (C) meal size and (D) meal number for standard chow for NTg (black bars) synphilin-1 mice (white bars) at the “obese” time-point. *<0.05, compared with non-transgenic mice.

In the older “obese” cohort, SP1 mice were significantly heavier than NTg controls and SP1 mice consumed significantly more chow than age-matched controls {Fig. 3A,t(11) = −2.302, p = 0.042}. Two-sample t-tests revealed that SP1 mice consumed significantly larger meals compared with the non-transgenic control mice {Figure 3B; (t(11) = −2.628, p = 0.024)}. In contrast, there was no significant group difference in meal number between SP1 and non-transgenic control mice {Figure 3C; (t) = 0.227, p = 0.824}.

### Pre-obese SP1 Mice Initiated Significantly More Trials to Sucrose and Licked More Vigorously to the Higher Sucrose Concentration

Increased intake and larger meal sizes may be driven by elevated orosensory stimulation. Thus, to assess orosensory responsivity, unconditioned licking responses to a concentration array of sucrose was measured in SP1 and NTg controls in a brief-access taste test. The brief-access taste procedure involves presenting a range of taste solution concentrations in short (5-s) trials across one session and thus minimizes the effect of postingestive cues. The procedure also allows for some segregation of the appetitive and consummatory components of ingestive behavior. The mouse’s approach behavior to the spout and initiating licking can be considered appetitive behavior. The licking response within a 5-s trial follows contact with the taste stimulus and can be considered consummatory behavior.

**Figure 4:**
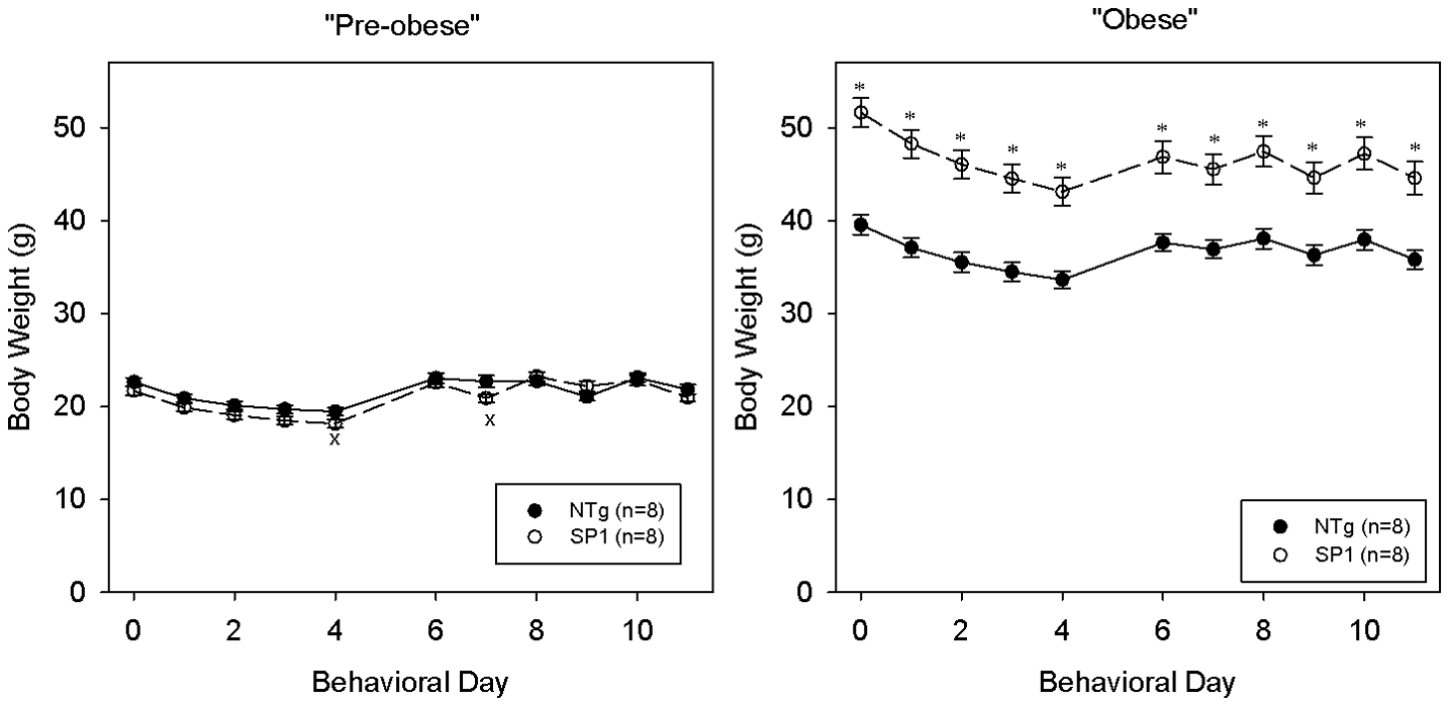
Body weight of pre-obese and obese SP1 mice. Body weight of mice as indicated were measured during testing days in the brief-access taste tests. NTg (black symbols) and synphilin-1 mice (white symbols). *p<0.05 compared with non-transgenic mice.

**Figure 5:**
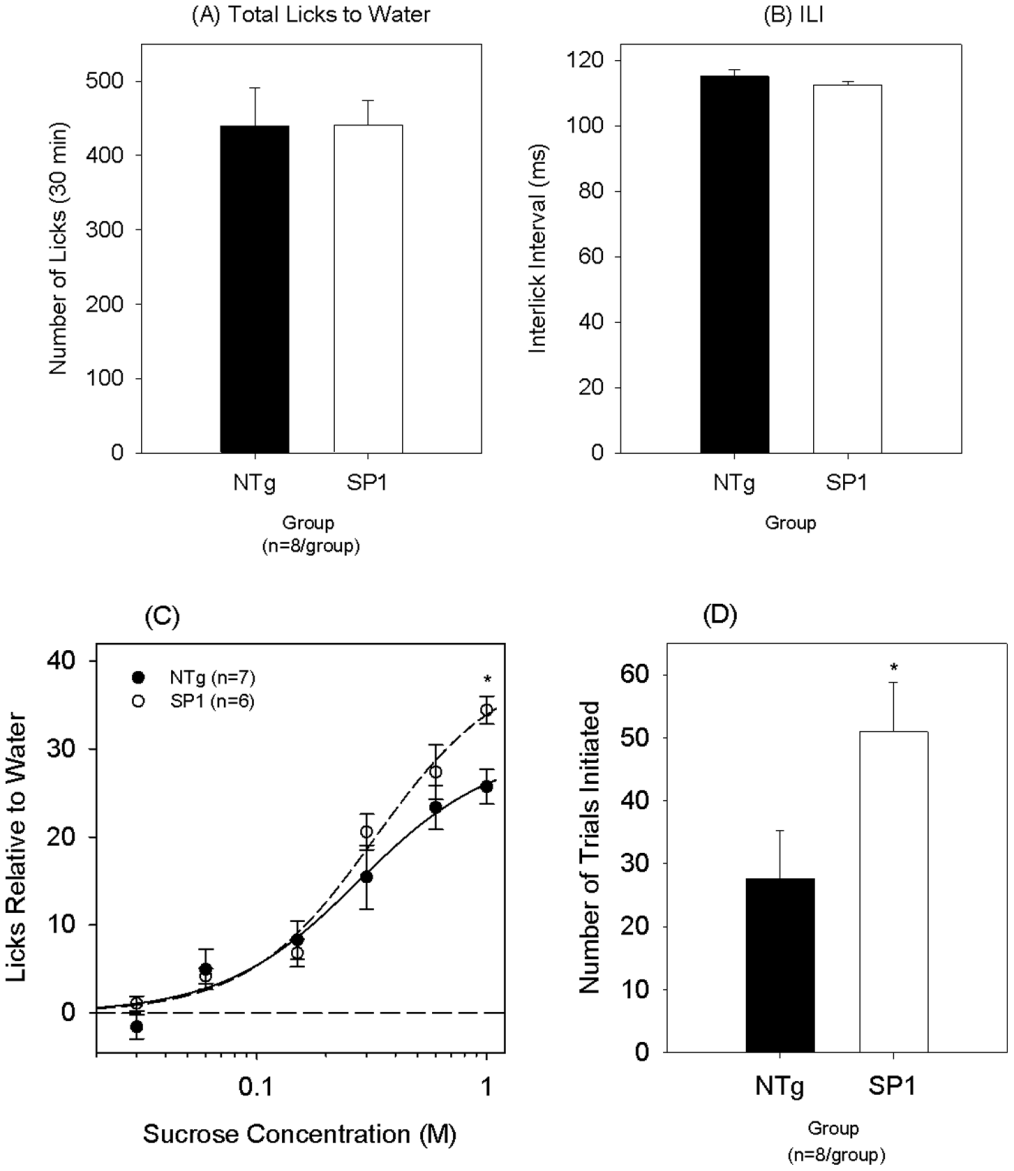
Licking response to sucrose in SP1 pre-obese mice. Mean (A) total licks and (B) interlick interval values to a stationary spout of water. (C) Licks relative to water across a sucrose concentration array and (D) number of trials initiated for NTg (black symbols) and synphilin-1 (white symbols) mice at the “pre-obese” time-point. *p<0.05, compared with non-transgenic mice.

At the pre-obese stage, a two-way ANOVA comparing body weights between the two groups across brief assess test days revealed no main effect of group {F(1,14) = 2.403, p = 0.143}, a main effect of day {F(10,140) = 214.831, p<0.001} and a significant interaction {F(10,140) = 4.242, p<0.001}. The SP1 group was significantly lighter on two testing days but this group difference did not reach Bonferonni correction (Figure 4). The groups did not differ in the total number of licks {Figure 5A; t(14) = 0.008, p = 0.994} or interlick interval (ILI) values to a stationary spout of water {Figure 5B; t(14) = −1.148, p = 0.270}. Two-way ANOVAs comparing sucrose licks relative to water values revealed no significant main effect of genotype {F(1,11) = 3.279, p = 0.098}, a main effect of concentration {F(5,55) = 81.148, p<0.001} and no significant interaction {F(5,55) = 1.996, p = 0.094} at the pre-obese stage. Post hoc t-tests revealed SP1 mice displayed significantly more licks to 1.0 M sucrose adjusted for water, compared to the controls {Figure 5C; t(11) = 3.492, p = 0.005 (p = 0.030)} that survived Bonferroni corrections. SP1 mice initiated significantly more trials to sucrose compared to the NTg controls {Figure 5D; t(14) = 2.140, p = 0.050}.

**Figure 6:**
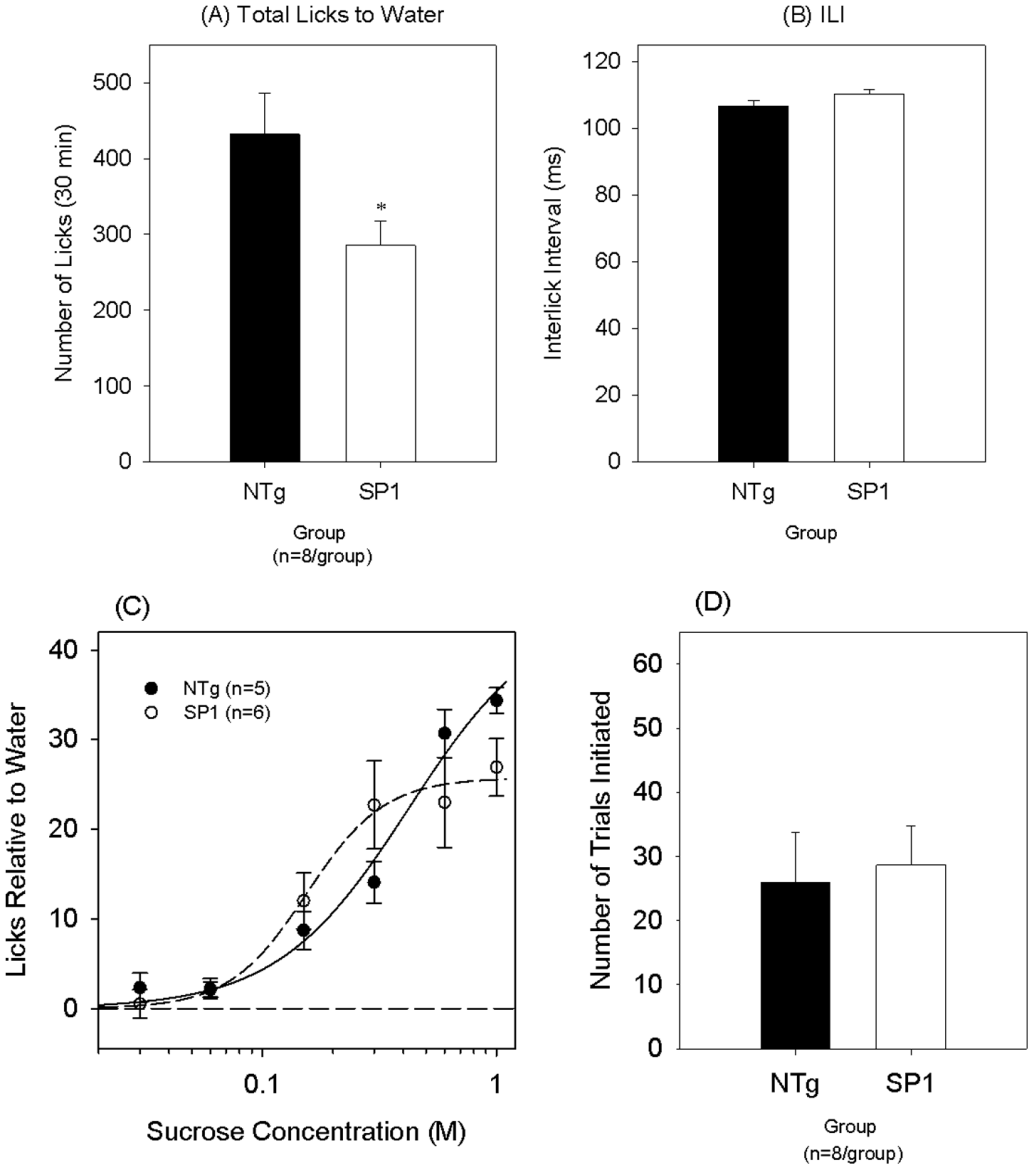
Licking response to sucrose of SP1 obese mice. Mean (A) total licks and (B) interlick interval values to a stationary spout of water. (C) Licks relative to water across a sucrose concentration array and (D) number of trials initiated for NTg (black symbols) and synphilin-1 (white symbols) mice at the “obese” time-point. *p<0.05, compared with non-transgenic mice.

At the obese stage (4 months of age), SP1 mice were significantly heavier than their NTg controls as confirmed by a main effect of group {F(1,14) = 26.586, p<0.001}, a main effect of day {F(10,140) = 88.759, p<0.001} and a significant interaction{(F(10,140) = 7.568, p<0.001}. To a stationary spout of water, total number of licks across a 30-min session was significantly lower in the SP1 group compared to the NTg controls {t(14) = −2.303, p = 0.037; Figure 6A} but there was no significant group difference in ILI values {t(14) = 1.799, p = 0.094; Figure 6B}. Two-way ANOVAs comparing sucrose licks relative to water values between the two groups revealed no main effect of group {F(1,10) = 0.090, p = 0.770}, revealed a main effect of concentration {F(5,50) = 51.297, p<0.001} and a significant interaction {F(5,50) = 3.331, p = 0.011}. Although a significant group × concentration interaction was revealed, two-sample t-tests conducted at each concentration did not reveal significant group differences (Figure 6C). There was no significant difference in the number of trials initiated during the sucrose testing sessions between SP1 and non-transgenic mice {t(14) = 0.274, 0.788; Figure 6D}.

## Discussion

In this study, we assessed various aspects of feeding behavior to further characterize the hyperphagia in SP1 mice. Meal pattern assessment revealed increases of meal size but no change of meal numbers in SP1 mice compared with non-transgenic controls. SP1 mice at pre-obese stage initiated significantly more trials to sucrose across the testing sessions and licked more vigorously to the highest concentration presented, compared to NTg controls. These findings indicate that the hyperphagia in SP1 mice is at least partly due to an increased appetitive behavioral component that may be indicative of enhanced food reward-related mechanisms.

Hyperphagia can be expressed as increases in meal size without significant changes in meal number in some obesity models [11], [12]. For instance, leptin and melanocortins that regulate food intake through modulating hypothalamic signaling pathways reduce food intake by altering the size of meal [13]–[17]. In SP1 mice, synphilin-1 is highly expressed in the arcuate and paraventricular nuclei of the hypothalamus, two central regions that play important roles in the controls of food intake [9]. Moreover, food deprivation significantly increases endogenous synphilin-1 expression in these two regions, suggesting a role for synphillin-1 in deprivation induced feeding [9]. Here, our data demonstrate that SP1 mice, whether tested at the younger “pre-obese” or older “obese” time points, had higher daily chaw intake compared to NTg controls. This increase of food intake was presented by increases in meal size without changing meal number. Increases in meal size of SP1 mice appear to be more robust in the older mice in which increased body weight was more pronounced. However, even in the “pre-obese” stage, there was a trend increase in meal size although it did not reach statistical significance.

When the diet was switched from standard chow to a 45% high-fat diet, both SP1 mice and NTg controls decreased meal number and increased meal size. Although SP1 mice showed significantly higher intake to standard chow compared to the NTg group, the group difference was no longer apparent when the mice were maintained on the high-fat diet. In the SP1 mice, total caloric intake was already relatively high on chow diet, thus it is plausible that in these mice, although high-fat diet elicits an increase in meal size, there is a ceiling effect preventing further increase in total daily calories.

Increased orosensory stimulation contributes greatly to human obesity and other obesity animal models [19], [30]. Our data showed that unconditioned licking responses to sucrose were similar across both groups at the “pre-obese” time point. However, the SP1 mice displayed more vigorous licking at the highest concentration and also initiated significantly more trials than their NTg counterparts giving evidence of increased ingestive behavioral components that likely contribute to increase in food intake in early development of obesity in SP1 mice. At the “obese” time point, these group differences were no longer apparent. Animals approach to the spout can be considered appetitive behavior while licking during a trial is behavior elicited by contact with the stimulus and thus can be considered consummatory. Collectively, these data indicate that consummatory behavior to a palatable liquid measured by unconditioned lick responses to sucrose are relatively normal in SP1 mice. Although measures of sensory threshold and affective responding can be experimentally differentiated [31], [32], these assessments are not necessarily mutually exclusive. The SP1 and NTg mice did not significantly differ in affective responding, therefore it suggest that synphilin-1 has little or no effect on orosensory responsivity to sucrose. Thus increased taste responsivity does not likely contribute to the hyperphagia observed in the SP1 mice. At the younger pre-obese time point, SP1 mice display elevated appetitive behavior towards trials to sucrose, suggesting that there may be increased motivation-related mechanisms that may contribute to the hyperphagia that eventually results in increased body weight in SP1 mice. Our data are similar to a previous report of a rat obesity model (OLETF rat), in which that alterations in taste response results in increase of meal size and food intake [33]–[36]. OLETF rats exhibit an increased response to sweet tastants but unaltered responses to other taste stimuli [34]. At the older obese time point at which SP1 mice are significantly heavier, this difference in appetitive behavior is no longer observed suggesting other underlying mechanism(s) may be in play that remains further studies.

At the older time point (obese stage), SP1 mice displayed significantly fewer licks to a stationary spout of water during a 30-min session when tested under a water-restricted condition. This was not observed at the “pre-obese” time point but only at the “obese” time point when the SP1 mice were significantly heavier. The two groups did not significantly differ in interlick-interval values, a measure of local lick rate [37], nor did the groups differ in licks to water during the 10-s trials thus suggesting the difference in water intake was not likely attributed to oromotor-related alterations or immediate consummatory responses to water. There are reports of increased intake or licks to water in other genetically obese rat models compared to lean controls [38], [39] and decreased water intake in diet-induced obesity rat studies [40]. It is unclear how the decreased licks to a stationary spout of water in the obese SP1 mice may be related to the alterations in ingestive behavior observed, which requires further investigation.

In summary, synphilin-1 overexpression induced hyperphagia appears to present as increases in meal size but not meal number. SP1 mice displayed an increased appetitive behavioral response to sucrose at pre-obese stage but no change with the addition of a high-fat diet. These findings indicate that synphilin-1-mediated hyperphagia is associated with meal size contribution factors, such as appetitive response to sucrose. These studies provide a novel insight into synphilin-1 regulating food intake behavior and energy homeostasis.
